# *N*-Benzyl-2-hy­droxy­ethanaminium cyanurate

**DOI:** 10.1107/S1600536813029383

**Published:** 2013-11-06

**Authors:** Carlos Abraham Contreras-Espejel, Marco A. García-Eleno, Ericka Santacruz-Juárez, Reyna Reyes-Martínez, David Morales-Morales

**Affiliations:** a Instituto de Química, Universidad Nacional Autónoma de México, Circuito exterior, Ciudad Universitaria, México, D.F., 04510, Mexico; b Universidad Politécnica de Tlaxcala Km. 9.5 Carretera Federal Tlaxcala-Puebla, Av. Universidad Politécnica No. 1 Xalcaltzingo, Tepeyanco, Tlaxcala, C.P., 90180, Mexico

## Abstract

In the cation of the title compound C_9_H_14_ON^+^·C_3_H_2_O_3_N_3_^−^, the benzyl­amine C—N bond subtends a dihedral angle of 78.3 (2)° with the phenyl ring. The cyanurate anion is in the usual keto-form and shows an r.m.s. deviation from planarity of 0.010 Å. In the crystal, the cyanurate anions form N—H⋯O hydrogen-bonded zigzag ribbons along [001]. These ribbons are crosslinked by the organocations *via* O—H⋯N and N—H⋯O hydrogen bonds, forming bilayers parallel to (010) which are held together along [010] by slipped π–π inter­actions between pairs of cyanurate anions [shortest contact distances C⋯C = 3.479 (2), O⋯N = 3.400 (2); centroid–centroid distance*=* 4.5946 (9) Å] and between cyanurate and phenyl rings [centroid–centroid distance = 3.7924 (12) Å, ring–ring angle = 11.99 (10)°].

## Related literature

For adducts of cyanuric acid, see: Sivashankar (2000 ▶); Ranganathan *et al.* (2000 ▶); Prior *et al.* (2013 ▶). For cyanurate and tri­thio­cyanurate salts, see: Krepps *et al.* (2001 ▶); Barszcz *et al.* (2006 ▶); Yang (2010 ▶); Nichol & Clegg (2006 ▶); Hou & Yang (2011 ▶); El-Gamel *et al.* (2008 ▶). For a common hydrogen-bond motif in cyanurates and tri­thio­cyanurates, see: Falvello *et al.* (1997 ▶); Sivashankar (2000 ▶); Hou & Yang (2011 ▶).
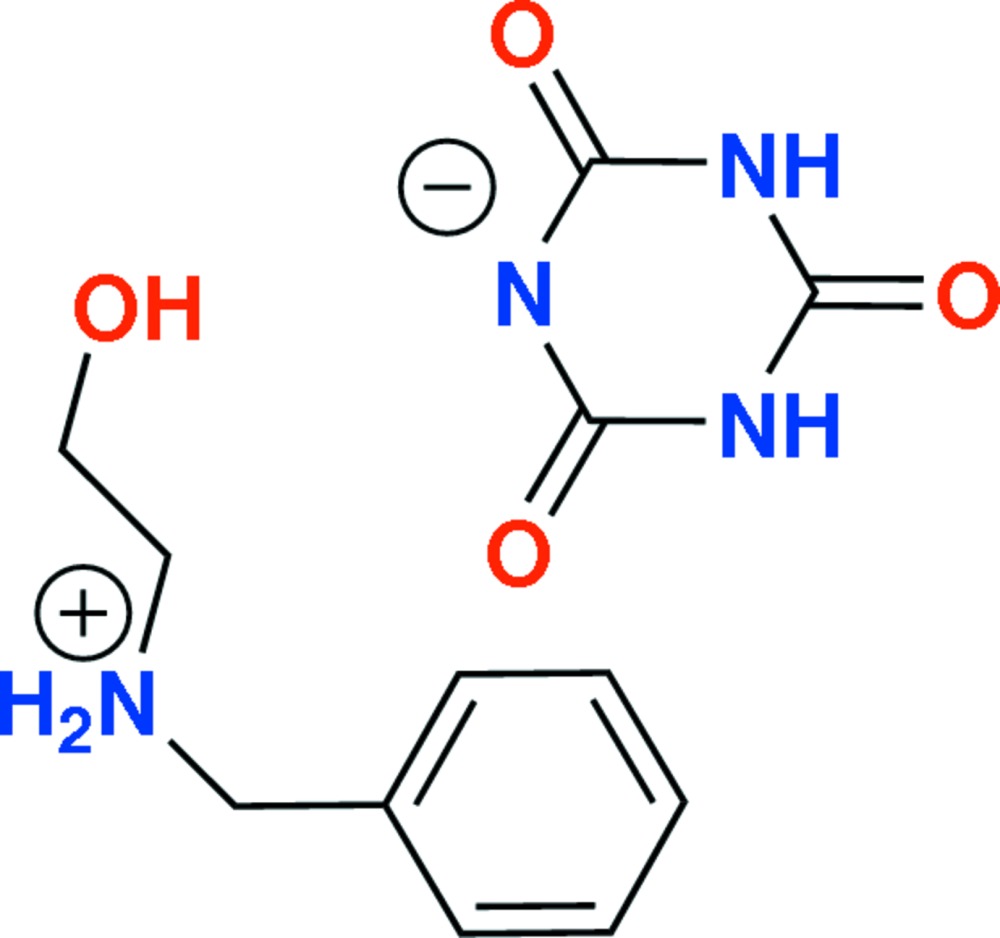


## Experimental

### 

#### Crystal data

C_9_H_14_NO^+^·C_3_H_2_N_3_O_3_^−^
*M* *_r_* = 280.29
Monoclinic, 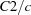

*a* = 21.0855 (3) Å
*b* = 14.0236 (2) Å
*c* = 10.0626 (1) Å
β = 115.474 (1)°
*V* = 2686.18 (6) Å^3^
*Z* = 8
Mo *K*α radiation
μ = 0.11 mm^−1^
*T* = 298 K
0.47 × 0.12 × 0.09 mm

#### Data collection

Bruker SMART APEX CCD diffractometer
Absorption correction: multi-scan (*SADABS*; Bruker, 2012 ▶) *T*_min_ = 0.68, *T*_max_ = 0.75
16557 measured reflections
2753 independent reflections
1913 reflections with *I* > 2σ(*I*)
*R*_int_ = 0.041

#### Refinement

*R*[*F* ^2^ > 2σ(*F* ^2^)] = 0.040
*wR*(*F* ^2^) = 0.115
*S* = 1.02
2753 reflections
197 parameters
H atoms treated by a mixture of independent and constrained refinement
Δρ_max_ = 0.20 e Å^−3^
Δρ_min_ = −0.17 e Å^−3^

### 

Data collection: *APEX2* (Bruker, 2012 ▶); cell refinement: *SAINT* (Bruker, 2012 ▶); data reduction: *SAINT*; program(s) used to solve structure: *SHELXS97* (Sheldrick, 2008 ▶); program(s) used to refine structure: *SHELXL97* (Sheldrick, 2008 ▶); molecular graphics: *ORTEP-3 for Windows* (Farrugia, 2012 ▶) and *DIAMOND* (Brandenburg, 2006 ▶); software used to prepare material for publication: *SHELXTL* (Sheldrick, 2008 ▶) and *PLATON* (Spek, 2009 ▶).

## Supplementary Material

Crystal structure: contains datablock(s) I, New_Global_Publ_Block. DOI: 10.1107/S1600536813029383/qk2061sup1.cif

Structure factors: contains datablock(s) I. DOI: 10.1107/S1600536813029383/qk2061Isup2.hkl

Additional supplementary materials:  crystallographic information; 3D view; checkCIF report

## Figures and Tables

**Table 1:** Hydrogen-bond geometry (Å, °)

*D*—H⋯*A*	*D*—H	H⋯*A*	*D*⋯*A*	*D*—H⋯*A*
N3—H3⋯O2^i^	0.921 (19)	1.841 (19)	2.7609 (17)	176.5 (16)
N5—H5⋯O4^ii^	0.879 (19)	1.97 (2)	2.8434 (17)	172.9 (17)
O1—H1⋯N1	0.91 (2)	1.82 (2)	2.7103 (17)	169 (2)
N2—H2*A*⋯O2^iii^	0.942 (19)	1.979 (19)	2.8612 (17)	155.2 (16)
N2—H2*B*⋯O1^iii^	0.939 (19)	2.003 (19)	2.835 (2)	146.6 (16)

Symmetry codes: (i) 
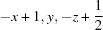
; (ii) 
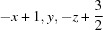
; (iii) 
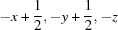
.

**Fig. 1.**
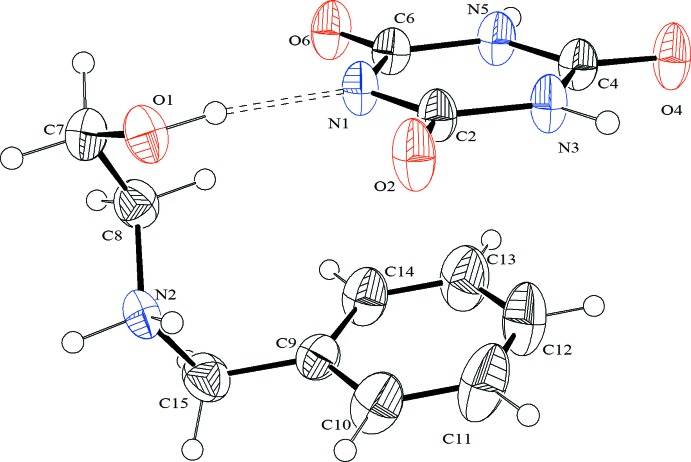
Asymmetric unit of the title compound with ellipsoids drawn at 40% probability.

**Fig. 2.**
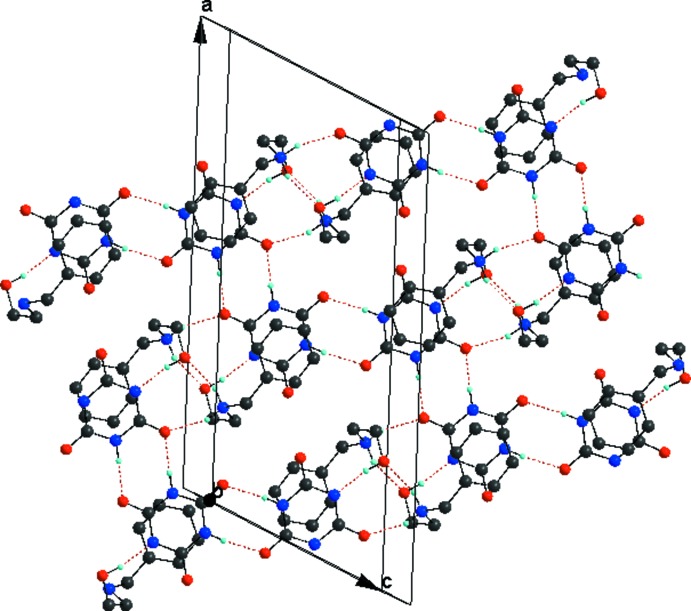
Hydrogen bond pattern in crystal structure of the title compound. Hydrogen bonds are shown as dashed lines.

## supplementary crystallographic information

### 1. Comment

Cyanuric acid and trithiocyanuric acid are based on planar six-membered rings and are used as building blocks of supramolecular assemblies held together via a variety of intermolecular hydrogen bonds. In undissociated form they contain in the solid state three hydrogen donors (N—H) and three hydrogen acceptors (O, S). In this form cyanuric acid generates adducts with pyridine (Sivashankar, 2000), 4,4'-bipyridyl (Ranganathan *et al.*, 2000) and melamine (Prior *et al.*, 2013). And both, cyanuric and trithiocyanuric acid are found in mono- and di-anionic form in various organic salts (Krepps *et al.*, 2001; Barszcz *et al.*, 2006), particularly as ammonium salts such as tripropylammonium (Yang, 2010), 1-dimethylammonio-8-dimethylaminonaphthalene (Nichol & Clegg, 2006), and guanidinium (El-Gamel *et al.*, 2008). Here we report the synthesis and crystal structure of the salt *N*-benzyl-2-hydroxyethanaminium cyanurate, [C_9_H_14_ON]^+^[C_3_H_2_O_3_N_3_]^-^.

The asymmetric unit of the title compound is formed by one molecule of the cyanurate anion and one molecule of the *N*-benzyl-2-hydroxyethanaminium cation (Fig. 1). The cyanurate anions are mutually linked *via* two pairs of centrosymmetric hydrogen bonds (N3—H3···O2^i^ and its inverse, N5—H5···O4^ii ^and its inverse) to form zig-zag ribbons along [001], a motif frequently encountered in cyanuric acid, cyanurate salts, and cyanurate metal complexes (Falvello *et al.*, 1997; Sivashankar, 2000; Hou *et al.*, 2011). Each *N*-benzyl-2-hydroxyethanaminium cation links two adjacent cyanurate zig-zag ribbons *via* the hydrogen bonds O1—H1···N1 and N2—H2A···O2^iii^ two form a 2-dimensional infinite bilayer parallel to (010) (Fig. 2). This bilayer is reinforced by the intercationic hydrogen bond N2—H2B···O1^iii^ and by an inclined π-π interaction between the cyanurate and the phenyl ring (*Cg*—*Cg* = 3.7924 (12) Å, ring-ring angle = 11.99 (10)°). Adjacent bilayers are held together along [010] by slipped π-π interactions between centrosymmetric pairs of cyanurate anions (shortest contact distances C4···C4(1-*x*,-*y*,1-*z*) = 3.479 (2) Å, O1···N3(1-*x*,-*y*,1-*z*) = 3.400 (2) Å; *Cg*—*Cg =* 4.5946 (9) Å). The incorporation of the *N*-benzyl-2-hydroxyethanaminium cation into the bilayer determines the conformation of the cation, which shows torsion angles of C10—C9—C15—N2 = 78.3 (2), C9—C15—N2—C8 = -168.71 (14), and C15—N2—C8—O1 = 59.3 (2)° for side chain atoms. The cyanurate ion is almost planar (r.m.s. and maximum deviations from planarity are 0.010 Å and 0.037 (15) Å (N5)).

### 2. Experimental

A mixture of triethylamine (1.5 g, 14.8 mmol) and 2-(benzylamino)ethanol (2 g, 13.5 mmol) was added slowly to methanolic solution of cyanuric chloride (0.83 g, 4.4 mmol) in an ice bath under stirring. The resulting solution was set to reflux for three days, and then allowed to cool down until the formation of a crystalline material was observed. The colourless crystalline material was filtered and washed with acetone and cold methanol.

### 3. Refinement

C-bonded H atoms were included in calculated position (C—H = 0.93 Å for aromatic H, and C—H = 0.97 Å for methylene H), and refined using a riding model with *U*_iso_(H) = 1.2 × *U*_eq_ of the carrier atoms. H atoms on N and O were located in a Fourier map and refined isotropically with *U*_iso_(H) = 1.2 × *U*_eq_(N) or 1.5 × *U*_eq_(O).

### Crystal data

**Table:**

C_9_H_14_NO^+^·C_3_H_2_N_3_O_3_^−^	*F*(000) = 1184
*M**_r_* = 280.29	*D*_x_ = 1.386 Mg m^−^^3^
Monoclinic, *C*2/*c*	Mo *K*α radiation, λ = 0.71073 Å
*a* = 21.0855 (3) Å	Cell parameters from 4964 reflections
*b* = 14.0236 (2) Å	θ = 2.5–26.0°
*c* = 10.0626 (1) Å	µ = 0.11 mm^−^^1^
β = 115.474 (1)°	*T* = 298 K
*V* = 2686.18 (6) Å^3^	Needle, colourless
*Z* = 8	0.47 × 0.12 × 0.09 mm

### Data collection

**Table:**

Bruker SMART APEX CCD diffractometer	2753 independent reflections
Radiation source: fine-focus sealed tube	1913 reflections with *I* > 2σ(*I*)
Graphite monochromator	*R*_int_ = 0.041
Detector resolution: 8.333 pixels mm^-1^	θ_max_ = 26.4°, θ_min_ = 1.8°
ω–scans	*h* = −26→26
Absorption correction: multi-scan (*SADABS*; Bruker, 2012)	*k* = −17→16
*T*_min_ = 0.68, *T*_max_ = 0.75	*l* = −12→12
16557 measured reflections	

### Refinement

**Table:**

Refinement on *F*^2^	Secondary atom site location: difference Fourier map
Least-squares matrix: full	Hydrogen site location: mixed
*R*[*F*^2^ > 2σ(*F*^2^)] = 0.040	H atoms treated by a mixture of independent and constrained refinement
*wR*(*F*^2^) = 0.115	*w* = 1/[σ^2^(*F*_o_^2^) + (0.0505*P*)^2^ + 1.1375*P*] where *P* = (*F*_o_^2^ + 2*F*_c_^2^)/3
*S* = 1.02	(Δ/σ)_max_ < 0.001
2753 reflections	Δρ_max_ = 0.20 e Å^−^^3^
197 parameters	Δρ_min_ = −0.17 e Å^−^^3^
0 restraints	Extinction correction: *SHELXL*, Fc^*^=kFc[1+0.001xFc^2^λ^3^/sin(2θ)]^-1/4^
Primary atom site location: structure-invariant direct methods	Extinction coefficient: 0.0026 (3)

### Special details

**Table:**

Geometry. All e.s.d.'s (except the e.s.d. in the dihedral angle between two l.s. planes) are estimated using the full covariance matrix. The cell e.s.d.'s are taken into account individually in the estimation of e.s.d.'s in distances, angles and torsion angles; correlations between e.s.d.'s in cell parameters are only used when they are defined by crystal symmetry. An approximate (isotropic) treatment of cell e.s.d.'s is used for estimating e.s.d.'s involving l.s. planes.

### Fractional atomic coordinates and isotropic or equivalent isotropic displacement parameters (Å^2^)

**Table:**

	*x*	*y*	*z*	*U*_iso_*/*U*_eq_	
N1	0.35291 (6)	0.12658 (10)	0.30864 (13)	0.0383 (3)	
O2	0.40501 (6)	0.13333 (10)	0.15312 (11)	0.0519 (4)	
C2	0.40936 (8)	0.12819 (12)	0.28065 (16)	0.0368 (4)	
N3	0.47580 (7)	0.12493 (11)	0.39537 (13)	0.0400 (4)	
H3	0.5148 (10)	0.1258 (12)	0.3762 (19)	0.048*	
C4	0.48802 (8)	0.11770 (12)	0.53885 (16)	0.0388 (4)	
O4	0.54804 (6)	0.11518 (10)	0.63875 (11)	0.0541 (4)	
N5	0.42916 (7)	0.11362 (11)	0.56146 (14)	0.0408 (4)	
H5	0.4334 (9)	0.1104 (12)	0.652 (2)	0.049*	
C6	0.36125 (8)	0.12036 (11)	0.44990 (16)	0.0364 (4)	
O6	0.31188 (6)	0.11963 (9)	0.48328 (13)	0.0506 (3)	
O1	0.22892 (6)	0.14867 (9)	0.06812 (13)	0.0496 (3)	
H1	0.2670 (12)	0.1384 (15)	0.154 (2)	0.074*	
N2	0.20910 (8)	0.34336 (11)	0.13192 (16)	0.0455 (4)	
H2A	0.1669 (10)	0.3632 (13)	0.054 (2)	0.055*	
H2B	0.2401 (10)	0.3266 (13)	0.091 (2)	0.055*	
C7	0.17466 (9)	0.17499 (14)	0.1081 (2)	0.0538 (5)	
H7A	0.1647	0.1220	0.1582	0.065*	
H7B	0.1323	0.1886	0.0198	0.065*	
C8	0.19408 (9)	0.26071 (13)	0.20647 (19)	0.0487 (5)	
H8A	0.1558	0.2766	0.2316	0.058*	
H8B	0.2352	0.2465	0.2969	0.058*	
C9	0.31829 (9)	0.40676 (12)	0.33215 (19)	0.0445 (4)	
C10	0.36875 (11)	0.41489 (17)	0.2800 (2)	0.0652 (6)	
H10	0.3559	0.4333	0.1830	0.078*	
C11	0.43848 (12)	0.3958 (2)	0.3712 (3)	0.0856 (8)	
H11	0.4722	0.4009	0.3351	0.103*	
C12	0.45783 (11)	0.3696 (2)	0.5138 (3)	0.0829 (8)	
H12	0.5047	0.3570	0.5750	0.099*	
C13	0.40907 (12)	0.36203 (19)	0.5662 (2)	0.0805 (7)	
H13	0.4225	0.3441	0.6635	0.097*	
C14	0.33930 (10)	0.38063 (15)	0.4766 (2)	0.0615 (6)	
H14	0.3062	0.3754	0.5143	0.074*	
C15	0.24244 (10)	0.42631 (13)	0.2327 (2)	0.0549 (5)	
H15A	0.2391	0.4828	0.1743	0.066*	
H15B	0.2172	0.4387	0.2918	0.066*	

### Atomic displacement parameters (Å^2^)

**Table:**

	*U*^11^	*U*^22^	*U*^33^	*U*^12^	*U*^13^	*U*^23^
N1	0.0261 (6)	0.0615 (9)	0.0268 (7)	−0.0001 (6)	0.0109 (5)	−0.0007 (6)
O2	0.0327 (6)	0.0998 (10)	0.0227 (6)	0.0031 (6)	0.0116 (5)	0.0033 (6)
C2	0.0296 (8)	0.0541 (10)	0.0249 (8)	0.0008 (7)	0.0099 (6)	−0.0006 (7)
N3	0.0254 (7)	0.0706 (10)	0.0246 (7)	0.0000 (6)	0.0112 (5)	0.0005 (6)
C4	0.0319 (8)	0.0575 (11)	0.0269 (8)	−0.0007 (7)	0.0126 (7)	−0.0008 (7)
O4	0.0298 (6)	0.1026 (11)	0.0256 (6)	−0.0008 (6)	0.0079 (5)	0.0018 (6)
N5	0.0328 (7)	0.0683 (10)	0.0222 (7)	−0.0014 (6)	0.0124 (6)	0.0005 (6)
C6	0.0320 (8)	0.0486 (10)	0.0303 (8)	−0.0014 (7)	0.0148 (7)	−0.0030 (7)
O6	0.0345 (6)	0.0849 (10)	0.0386 (6)	−0.0021 (6)	0.0215 (5)	−0.0028 (6)
O1	0.0426 (7)	0.0633 (8)	0.0348 (7)	0.0070 (6)	0.0090 (5)	0.0004 (6)
N2	0.0357 (8)	0.0533 (9)	0.0366 (8)	0.0066 (7)	0.0052 (6)	0.0049 (7)
C7	0.0346 (9)	0.0589 (12)	0.0596 (12)	−0.0028 (8)	0.0123 (8)	0.0033 (9)
C8	0.0418 (10)	0.0574 (12)	0.0499 (10)	0.0061 (8)	0.0225 (8)	0.0072 (8)
C9	0.0467 (10)	0.0429 (10)	0.0424 (10)	−0.0047 (8)	0.0179 (8)	−0.0090 (8)
C10	0.0614 (13)	0.0930 (16)	0.0436 (11)	−0.0183 (11)	0.0248 (10)	−0.0094 (10)
C11	0.0518 (13)	0.139 (2)	0.0730 (16)	−0.0237 (14)	0.0335 (12)	−0.0226 (15)
C12	0.0456 (12)	0.123 (2)	0.0627 (15)	−0.0081 (12)	0.0067 (11)	−0.0152 (14)
C13	0.0634 (14)	0.123 (2)	0.0427 (12)	−0.0133 (14)	0.0113 (11)	0.0012 (12)
C14	0.0524 (11)	0.0904 (16)	0.0414 (10)	−0.0084 (10)	0.0197 (9)	−0.0079 (10)
C15	0.0548 (11)	0.0468 (11)	0.0568 (11)	0.0088 (9)	0.0180 (9)	−0.0008 (9)

### Geometric parameters (Å, º)

**Table:**

N1—C2	1.3361 (19)	C7—H7A	0.9700
N1—C6	1.3580 (19)	C7—H7B	0.9700
O2—C2	1.2487 (18)	C8—H8A	0.9700
C2—N3	1.3807 (19)	C8—H8B	0.9700
N3—C4	1.3569 (19)	C9—C14	1.375 (3)
N3—H3	0.921 (19)	C9—C10	1.379 (3)
C4—O4	1.2324 (18)	C9—C15	1.503 (2)
C4—N5	1.3559 (19)	C10—C11	1.384 (3)
N5—C6	1.392 (2)	C10—H10	0.9300
N5—H5	0.879 (19)	C11—C12	1.363 (3)
C6—O6	1.2237 (18)	C11—H11	0.9300
O1—C7	1.415 (2)	C12—C13	1.346 (3)
O1—H1	0.91 (2)	C12—H12	0.9300
N2—C8	1.487 (2)	C13—C14	1.380 (3)
N2—C15	1.504 (2)	C13—H13	0.9300
N2—H2A	0.942 (19)	C14—H14	0.9300
N2—H2B	0.939 (19)	C15—H15A	0.9700
C7—C8	1.498 (3)	C15—H15B	0.9700
			
C2—N1—C6	119.72 (13)	N2—C8—H8A	109.6
O2—C2—N1	122.64 (14)	C7—C8—H8A	109.6
O2—C2—N3	117.44 (14)	N2—C8—H8B	109.6
N1—C2—N3	119.92 (13)	C7—C8—H8B	109.6
C4—N3—C2	123.52 (13)	H8A—C8—H8B	108.1
C4—N3—H3	116.4 (11)	C14—C9—C10	118.30 (18)
C2—N3—H3	120.0 (11)	C14—C9—C15	121.32 (17)
O4—C4—N5	123.70 (14)	C10—C9—C15	120.38 (17)
O4—C4—N3	121.90 (14)	C9—C10—C11	120.35 (19)
N5—C4—N3	114.39 (14)	C9—C10—H10	119.8
C4—N5—C6	124.08 (13)	C11—C10—H10	119.8
C4—N5—H5	119.0 (11)	C12—C11—C10	120.1 (2)
C6—N5—H5	116.8 (11)	C12—C11—H11	119.9
O6—C6—N1	123.05 (14)	C10—C11—H11	119.9
O6—C6—N5	118.67 (14)	C13—C12—C11	120.0 (2)
N1—C6—N5	118.28 (13)	C13—C12—H12	120.0
C7—O1—H1	105.3 (14)	C11—C12—H12	120.0
C8—N2—C15	113.71 (14)	C12—C13—C14	120.6 (2)
C8—N2—H2A	108.8 (11)	C12—C13—H13	119.7
C15—N2—H2A	109.5 (11)	C14—C13—H13	119.7
C8—N2—H2B	111.4 (12)	C9—C14—C13	120.62 (19)
C15—N2—H2B	106.1 (11)	C9—C14—H14	119.7
H2A—N2—H2B	107.0 (15)	C13—C14—H14	119.7
O1—C7—C8	111.87 (14)	C9—C15—N2	111.28 (14)
O1—C7—H7A	109.2	C9—C15—H15A	109.4
C8—C7—H7A	109.2	N2—C15—H15A	109.4
O1—C7—H7B	109.2	C9—C15—H15B	109.4
C8—C7—H7B	109.2	N2—C15—H15B	109.4
H7A—C7—H7B	107.9	H15A—C15—H15B	108.0
N2—C8—C7	110.42 (14)		
			
C6—N1—C2—O2	179.62 (16)	O1—C7—C8—N2	59.3 (2)
C6—N1—C2—N3	−0.9 (2)	C14—C9—C10—C11	0.8 (3)
O2—C2—N3—C4	−178.86 (16)	C15—C9—C10—C11	−179.3 (2)
N1—C2—N3—C4	1.7 (3)	C9—C10—C11—C12	−0.6 (4)
C2—N3—C4—O4	−179.83 (16)	C10—C11—C12—C13	0.2 (4)
C2—N3—C4—N5	0.2 (2)	C11—C12—C13—C14	−0.1 (4)
O4—C4—N5—C6	177.24 (17)	C10—C9—C14—C13	−0.7 (3)
N3—C4—N5—C6	−2.7 (2)	C15—C9—C14—C13	179.37 (19)
C2—N1—C6—O6	179.16 (15)	C12—C13—C14—C9	0.4 (4)
C2—N1—C6—N5	−1.5 (2)	C14—C9—C15—N2	−101.8 (2)
C4—N5—C6—O6	−177.10 (16)	C10—C9—C15—N2	78.3 (2)
C4—N5—C6—N1	3.5 (2)	C8—N2—C15—C9	74.5 (2)
C15—N2—C8—C7	−168.71 (14)		

### Hydrogen-bond geometry (Å, º)

**Table:**

*D*—H···*A*	*D*—H	H···*A*	*D*···*A*	*D*—H···*A*
N3—H3···O2^i^	0.921 (19)	1.841 (19)	2.7609 (17)	176.5 (16)
N5—H5···O4^ii^	0.879 (19)	1.97 (2)	2.8434 (17)	172.9 (17)
O1—H1···N1	0.91 (2)	1.82 (2)	2.7103 (17)	169 (2)
N2—H2*A*···O2^iii^	0.942 (19)	1.979 (19)	2.8612 (17)	155.2 (16)
N2—H2*B*···O1^iii^	0.939 (19)	2.003 (19)	2.835 (2)	146.6 (16)

Symmetry codes: (i) −*x*+1, *y*, −*z*+1/2; (ii) −*x*+1, *y*, −*z*+3/2; (iii) −*x*+1/2, −*y*+1/2, −*z*.
